# Assessment of Parental Knowledge towards Space Maintainer as an Essential Intervention after Premature Extraction of Primary Teeth

**DOI:** 10.3390/healthcare10061057

**Published:** 2022-06-07

**Authors:** Amel Ali, Mamata Hebbal, Nada Aldakheel, Norah Al Ghamdi, Elzahraa Eldwakhly

**Affiliations:** 1 Department of Pediatric Dentistry and Oral Public Health, Faculty of Dentistry, Alexandria University, Alexandria 21526, Egypt; d.amelmahmoud@hotmail.com; 2 Department of Preventive Dental Sciences, College of Dentistry, Princess Nourah bint Abdulrahman University, P.O. Box 84428, Riyadh 11671, Saudi Arabia; mihebbal@pnu.edu.sa; 3 General Dentist, Riyadh 13216, Saudi Arabia; aldknada124@gmail.com (N.A.); n-alghamdy@hotmail.com (N.A.G.); 4 Department of Clinical Dental Sciences, College of Dentistry, Princess Nourah bint Abdulrahman University, P.O. Box 84428, Riyadh 11671, Saudi Arabia

**Keywords:** children oral health, parent awareness, premature tooth loss, space maintainer

## Abstract

This study assessed parents’ knowledge about space maintainers as an interceptive strategy after premature loss of primary teeth. Methods and Material: A cross-sectional questionnaire-based descriptive study was conducted on a sample of 600 parents; 456 (76%) were females, and 144 (24%) were males, recruited from an outpatient dental clinic at Princess Nourah bint Abdulrahman University, Riyadh, Saudi Arabia. The questionnaire consisted of two sections; the first section collected sociodemographic data of participants, and the second section gathered parental knowledge of space maintainers. The research ethics committee granted ethical approval, and informed consent was obtained before participation. Results: 600 parents participated in this study. The total mean knowledge score regarding space maintainers was 7.97 ± 3.38. The mean knowledge score was 7.06 ± 2.99 for male parents and 8.26 ± 3.45 for female parents. The difference in the mean knowledge score between female and male parents was statistically significant (*p* < 0.001). Additionally, there was a statistically significant difference in the knowledge score and parents’ age groups, with parents having the highest knowledge in the 30–40 age group (*p* = 0.0197). There was a positive correlation between knowledge scores and the number of children. Parents with one child showed less knowledge than those with two to four children (*p* = 0.0121). Conclusions: Participants’ knowledge regarding space maintainers was 49.8%, which is considered inadequate. It was deemed essential to increase knowledge among parents about space maintainers as an interceptive approach after premature loss of primary teeth.

## 1. Introduction

Dental caries is a major oral health problem among children. According to the United States Surgeon General’s report on oral health, dental caries is the single most common chronic childhood disease and the most prevalent unmet health need among children [1].

Epidemiological studies showed that early childhood caries is still prevalent worldwide and frequently causes untimely extractions despite the implemented preventive programs as a practical approach for preventing caries-related premature tooth loss in children and conserving relevant arch length [2,3]. A recent prospective birth cohort study evaluated a prevention program’s effect on early childhood caries inhibition and elimination of the need for orthodontic treatment. Children enrolled in the prevention program were compared with children of the same age whose parents decided not to participate in the program. All five-year-old participating children were examined, dental caries was scored, and impressions were taken for children with prematurely lost teeth to evaluate space impairment. Consequently, children enrolled in the prevention program showed significantly lower caries prevalence, whereas orthodontic analysis found a higher prevalence of space loss following premature tooth extraction in the children of parents who decided not to participate in the prevention program [4]. Children receiving continuous dental care starting at birth (dental home and anticipatory guidance) reported better oral health with less premature deciduous teeth loss and more intact primary arches, leading to preservation of space for permanent teeth and maintenance of arch length. Various factors, such as neglected oral hygiene, frequent snacking and a high-carbohydrate diet, increase susceptibility to dental caries and result in premature extractions of primary teeth, thereby demanding the placement of space maintainers [2,3]. Benefits of space maintainers include guided eruption of the permanent teeth and the avoidance of complex orthodontic treatment afterwards [5]. A previous study determined whether the premature loss of the deciduous second molar results in a clinically relevant loss of space and arch length reduction. They measured the extraction and control sides and analyzed the premolars’ space loss and eruption stage in three weeks. They concluded that space loss on the extraction side was statistically significant, thereby emphasizing the use of space maintainers at the earliest stage after extraction [6].

Parental knowledge about children’s dental care and oral hygiene influences their overall health and quality of life [3]. Proper education for patient/parents regarding the care of the primary and mixed dentition is the responsibility of the pediatric dentist. Physiologically, when lost, primary teeth will sooner be replaced by permanent teeth [7]. Premature loss of primary teeth causes harm to the occlusion and to the eruption of the permanent teeth. The dentition might be disturbed because of certain conditions, such as severe early childhood caries, traumatic injuries, dental anomalies or systemic illnesses that prompt untimely loss of primary dentition [3,8].

Additionally, early primary teeth loss negatively influences the dental arches and may lead to mesial drifting of posterior molars, distal drifting of anterior teeth and over eruption of opposing teeth [9,10]. There is a strong tendency for the mandibular permanent first molar to drift medially even before eruption. Extra space loss could occur if teeth actively erupt adjacent to the space left by the premature primary tooth loss. When teeth are lost prematurely, the forces that hold the tooth in its proper position are no longer in equilibrium, leading to the tipping of adjacent teeth into the open space and blocking of the unerupted permanent tooth. Placing a space maintainer balances these forces and allows for normal eruption of the permanent tooth by holding the adjacent teeth in position during the eruption stage [7,11].

The prevention of malocclusion incidence is realized by utilizing space management as an interceptive approach to maintain the space created by the premature loss of deciduous teeth preceding permanent teeth eruption [12,13]. The appropriate timing is crucial when planning for space management, and delayed intervention is sometimes useless since it is proven that space loss might occur within a few days after an extraction [5]. Space closure occurs for six months following the extraction, and sometimes within days. The best approach is to insert a space maintainer immediately after extraction [7,11]. Regular follow-up appointments and maintenance protocol are mandatory for a proper approach since a neglected appliance may deteriorate an existing space [7]. Zhang et al. used a self-designed questionnaire to investigate the essential basic information and related problems of 36 parents whose children received space loss treatment. The findings were that parents paid more attention to treatment than to prevention. Additionally, most parents (72.22%) did not know the harmful effect of space loss nor the necessity of treatment after premature extraction (58.33%) [14].

Few published studies assessed the knowledge and awareness regarding space maintainers. However, these studies assessed knowledge either among dentists and general practitioners or among the parents in private pediatric dentistry clinics, which targets highly educated parents who can afford the fees of private services [10,12]. Hence, the current study selected a government setup to represent a broader patient population to assess parents’ knowledge of space maintainers and compare the knowledge among different sociodemographic parent groups.

## 2. Materials and Methods

The present study was a cross-sectional questionnaire-based study conducted on a sample size of 600 parents recruited from the outpatient dental clinic at Princess Nourah bint Abdulrahman University (PNU), Riyadh, Saudi Arabia. Ethical approval to conduct the study was obtained from the PNU ethics committee with IRB registration number (H-01-R-059).

### 2.1. Sample Size Estimation

The sample size estimation was based on a previous study [10], where the percentage of correct responses ranged from 40% to 74% for different questions, and the average percentage of correct responses was considered for the sample size calculations based on the following formula:

n = (1.96)^2^ × pq/d^2^,

{p = 57, q = 43, d = 4}

The result was 588, rounded to 600 participants. 

### 2.2. Development of the Questionnaire

When the research was planned, related articles were reviewed for the questionnaire [2,3,10]. None of the articles had a questionnaire which would exactly meet the study objectives. Therefore, we decided to develop a new questionnaire and test its validity and reliability.

A 14-items questionnaire was prepared with closed-ended questions. Two authors designed the questionnaire in English, and seven endodontists reviewed the questionnaire and identified items necessary for retention based on content validity assessment. Two individual experts translated the questionnaire. The first expert translated the questionnaire from English into Arabic, and the second expert back-translated it to English. The back-translated English version was compared with the original English version to confirm the similarity of words without changing the meaning. Before implementing the study, the final questionnaire was given to five participants to check their comprehension and understanding of the wordings. Later, six randomly selected participants were asked to answer the questionnaire twice at two different time intervals to check the reliability.

### 2.3. Details of the Questionnaire

The questionnaire consisted of two sections. The first section had four questions concerning the sociodemographic data of the participants. The second section had ten questions that collected parental knowledge about space maintainers and their children’s dental experience. Three questions from the second section were related to the child’s dental visits and management of any previous experience with missing teeth. The next seven questions were related to parental knowledge about space maintainers. The parental knowledge score was calculated by quantifying the response options from zero to two or zero to three based on the importance of the response. For example, for a particular question, a score of two quantified the correct response, one quantified the “do not know” response, and a score of zero quantified the wrong response. This scoring decision was based on the expert opinion that the response of “do not know” is better than having wrong knowledge. When the maximum scores of all the questions were added, it was 16. Thus, a participant could get a final knowledge score ranging from 0 to 16. Zero was the worst total mean knowledge score, and 16 was the best total mean knowledge score.

### 2.4. Data Collection Procedure

Data were collected using Google Forms. The purpose of the study was explained to parents waiting in the dental outpatient clinic, and informed consent was obtained before participation in the study. The parents were asked to mark the most appropriate answer from the given options for each question, and sufficient time was given to complete the questionnaire. Any doubts were cleared without providing a hint to the correct answer. 

### 2.5. Statistical Analysis

The Excel sheet was generated from Google Forms and subjected to statistical analysis using JMP 14.2.0 (SAS Institute Inc. 100 SAS Campus Drive Cary, NC, USA). Descriptive statistics were calculated in terms of frequency, percentages for categorical data, mean and standard deviation for quantified knowledge scores. Unpaired *t*-test and ANOVA were used to find any difference between mean awareness level and demographic variables. The significance level was set at *p* < 0.05.

## 3. Results

A total of 600 parents participated in this study, of whom 456 (76%) were females, and 144 (24%) were males. The majority of the participants were in the age range of 31–45 years (59%), 68% had completed university studies as their highest education, and 54% had two to four children; see Table 1.

Seventy-seven percent of the participants indicated that their children had visited the dentist, and 37% of children had lost teeth because of caries, trauma or other reason. More than 50% of the participants selected either a wrong answer or the “I do not know option” to the knowledge questions; see Table 2 and Table 3.

The total mean knowledge score regarding space maintainers was 7.97 ± 3.38. The mean knowledge score for males was 7.06 ± 2.99 and 8.26 ± 3.45 for females, and the difference in their mean knowledge score was statistically significant (*p* < 0.001). There was a statistically significant difference in the knowledge score regarding age group, with the 30–40-year-old parents having the highest knowledge (*p* = 0.0197). There was a statistically significant difference in the knowledge score and the number of children in the family. Parents with one child showed less knowledge than those with two to four children (*p* = 0.0121). The knowledge regarding space maintainers among the participants was 49.8%, which was calculated based on the mean knowledge score (7.97) and maximum possible score (16); see Table 4.

## 4. Discussion

Worldwide studies on dental caries have proved that up to 65% of preschoolers are affected by caries despite all efforts toward caries prevention. Consequently, this counts for as many as 92.6% of the premature extractions of deciduous molars in children of less than six years of age [15]. Considerable research in Saudi Arabia reports an alarmingly high percentage of early childhood caries ranging between 82% and 90% [16].

In the present study, when parents were asked if they knew about the harms resulting from the early loss of deciduous teeth, 25% replied “No,” and about 29% were unaware. Meanwhile, when asked if any of their children experienced teeth loss due to caries or trauma, 37% replied “Yes,” confirming the importance and need to conduct this survey. The present study findings are in accordance with those of Setty and Srinivasan, who reported that 39% of parents were aware of the importance of deciduous teeth and their role in preserving space [17]. 

In the present study, when asking parents if they had ever been to a pediatric dentist, about 77% answered “Yes,” which means that they knew the importance of regular dental visits but still had a shortage of knowledge about preventive/interceptive dentistry. Concurrently, most participants had no idea about the frequent maintenance appointments after insertion of the space maintainer nor how to manage sudden eruption and accidental breakage. This lack of knowledge emphasizes the importance of instructing the patients and their families about maintaining the arch integrity after premature loss of teeth, as it is the leading cause of malocclusion among children.

A similar questionnaire survey was conducted in India among 100 parents with children between 2 and 16 years. It was found that 65% of parents reported visiting dentists only when the child complained about pain, and they thought that treating primary teeth was not very important, as it would eventually exfoliate Additionally, parents were unaware of the various treatment modalities available for space management after the extraction of primary teeth, which agrees with the present study’s findings [18].

Nagarajappa et al. reported that the actual disease and the perceived need for treatment significantly correlated with the parent’s perceptions and awareness of their children’s oral health. If proper knowledge is established, preventive care and space maintainer demand will also improve [19].

On the other hand, Alshehri and Nasim assessed parents’ knowledge and awareness about their infants’ oral healthcare. They revealed that only 25.33% of participants had good knowledge about their children’s oral health [20]. A similar questionnaire-based study showed that the level of awareness of parents of the factors related to space maintainers for children is inadequate [21]. 

A recent cross-sectional questionnaire-based study was implemented to assess parents’ awareness of space maintainers in Al-Kharj, Saudi Arabia. As per the results of the current study, it was found that around 82.1% of parents were not aware of space maintainers, nor did they receive any information about their benefits. Similarly, 73.7% did not know when to use space maintainers. Confirming our findings, knowledge of space maintainers and space management among Saudi parents is deficient [22]. 

In the present study, parents who reported that the dentist was their source of knowledge about space maintainers accounted for 19% of participants, which is considered a low percentage. In contrast to previous studies, the current study responses were quantified, and hence an overall mean knowledge score could be calculated, which might facilitate the comparison. No statistically significant difference was found between parents’ education levels and their knowledge about the importance and function of space maintainers. Parents aged 30 and above were more knowledgeable about premature teeth loss and its deleterious effects. Parents with more children had better knowledge, but this is considered self-gained knowledge, not an educational dental service. The current results show that the community still lacked knowledge about the space maintainer as an interceptive strategy after premature extraction of primary teeth, which was proved by the total knowledge percentage of 49.8%. This finding is in accordance with a previous similar study analyzing parental awareness about different malocclusion problems commonly arising during childhood [23]. Pediatric dentists should contribute toward: raising awareness, helping parents understand what to expect during the child’s current and approaching stages of development and providing personalized instructions and education programs. 

## 5. Limitations of the Study

The current study was conducted among the outpatient clinic attendees of a single dental institute; the sample may not represent the whole population. Researchers cannot rule out the possibility of overestimating the knowledge due to this being a hospital-based study. Hence, the results should be generalized with caution. 

## 6. Conclusions

It was concluded that parents’ knowledge about space maintainer as an interceptive strategy was apparently deficient, and that female parents in the older age group with more than one child were more knowledgeable when compared to other parent groups. Pediatric dentists must raise knowledge among parents regarding space maintainers and their longevity during regular dental visits in order to keep the integrity and minimize occlusal discrepancies of dental arches. 

## Figures and Tables

**Table 1:** Distribution of study participants according to sociodemographic variables.

		Number (%)
Gender	Female Male	456 (76) 144 (24)
Age	20–30 31–45 46 and above	124 (21) 352 (59) 124 (20)
Educational level	Primary Intermediate Secondary University	13 (2) 49 (8) 132 (22) 406 (68)
No. of children	1 2–4 More than 4	110 (18) 324 (54) 166 (28)

**Table 2:** Distribution of study participants according to responses for questions with an answering scheme of “Yes or No”.

Question/Answer	Yes	No
	Number (%)	Number (%)
Q1: Has your child ever visited the dentist?	464 (77)	124 (20)
Q2: Has your child ever lost his teeth because of caries or trauma or any other reason?	224 (37)	335 (56)
Q3: Does early loss of primary teeth will harm the permanent teeth	277 (46)	149 (25)
Q4: Did your child’s dentist ever explain how it is important to use a space maintainer after extraction?	101 (17)	407 (68)
Q5: Have any of your child had a space maintainer?	63 (11)	446 (74)
Q6: Does a child with a space maintainer need regular visits to the dentist	207 (35)	44 (7)

**Table 3:** Distribution of study participants according to responses for questions with answering scheme of different options.

Questions	Options	Number (%)
Q7: How do you know about the space maintainer?	Dentist	167(16)
	Family and relatives	58 (10)
	Social media	37 (6)
	I Don’t know	390 (65)
Q8: What should you do if the space maintainer breaks?	I leave it	26 (3)
	I will immediately go to the dentist	167 (16)
	I Don’t know	407 (81)
Q9: What should you do when the permanent teeth are erupting while the child still wearing the space maintainer?	I will immediately go to the dentist	176 (29)
	Wait for the Next visit	16 (3)
	I Don’t care	42 (7)
	I Don’t know	366 (61)
Q10: What type of food is prohibited for children wearing a space maintainer?	Soft drinks	47 (8)
	Vegetables and fruit	12 (2)
	Candies	95 (16)
	All above	102 (17)
	I Don’t know	344 (57)

**Table 4:** Comparison of mean knowledge score of participants according to demographic variables.

Variables		Mean	SD	*p* Value (*t* Test/ANOVA)
	Total mean	7.97	3.38	
Gender	Female	8.26	3.45	<0.001
	Male	7.06	2.99	
Age	20–30	8.02	3.15	0.0197
	31–45	8.22 *	3.61	
	46 and above	7.23 *	2.8	
Education level	Primary	6.92	2.66	0.1794
	Internment	7.28	2.53	
	Secondary	7.78	3.25	
	University	8.15	3.52	
No. of children	1	7.26 *	2.69	0.0121
	2–4	8.32 *	3.55	
	More than 4	7.77	3.4	

* Post hoc significance between groups.

## Data Availability

The data presented in this study are available on request from the corresponding author.
